# Cardiac MR detects the progression of impaired myocardial perfusion reserve in a mouse model of obesity-related cardiomyopathy

**DOI:** 10.1186/1532-429X-17-S1-P82

**Published:** 2015-02-03

**Authors:** Nivedita K Naresh, Joshua T Butcher, Xiao Chen, Brian H Annex, Brant E Isakson, Frederick H Epstein

**Affiliations:** 1 Department of Biomedical Engineering, University of Virginia, Charlottesville, VA, USA; 2 Robert M. Berne Cardiovascular Research Center, University of Virginia, Charlottesville, VA, USA; 3 Department of Molecular Physiology and Biological Physics, University of Virginia, Charlottesville, VA, USA; 4 Department of Cardiovascular Medicine, University of Virginia, Charlottesville, VA, USA; 5 Department of Radiology, University of Virginia, Charlottesville, VA, USA

## Background

Obesity has become increasing prevalent in western society and is associated with increased risk of heart failure [1]. Increased body weight is independently associated with impaired myocardial perfusion reserve (MPR), even in the absence of obstructive coronary artery disease [2]. Mouse models can elucidate molecular mechanisms that underlie cardiovascular disease. In this study, we used *in vivo* cardiac MRI in a mouse model of diet-induced obesity to establish the time course of MPR and we further used *ex vivo* histological and vascular reactivity studies to elucidate factors underlying the MRI results.

## Methods

Six week old C57Bl/6 mice fed a high-fat diet (HFD) (n = 9) and age-matched C57Bl/6 mice fed a low-fat diet (Control) (n = 9) were imaged at 7T. Imaging was performed at 6, 12, 18 and 24 weeks post-diet. Mice were anesthetized with 1.25% isoflurane and maintained at 36±1°C during MRI. The MRI protocol included multi-slice cine imaging to assess ejection fraction, left-ventricular (LV) mass, LV wall thickness, and LV volumes, and first-pass imaging at rest and with the vasodilator Regadenoson (0.1 µg/g body weight) to quantify MPR. At 25 weeks post-diet, blood pressure was measured non-invasively. Vascular reactivity of isolated coronary arterioles was assessed using cumulative dose responses to adenosine and acetylcholine in a sub-group of the mice. Histology of the aorta detected the presence or absence of systemic atherosclerosis, and myocardial capillary density was quantified.

## Results

**Figure 1:**
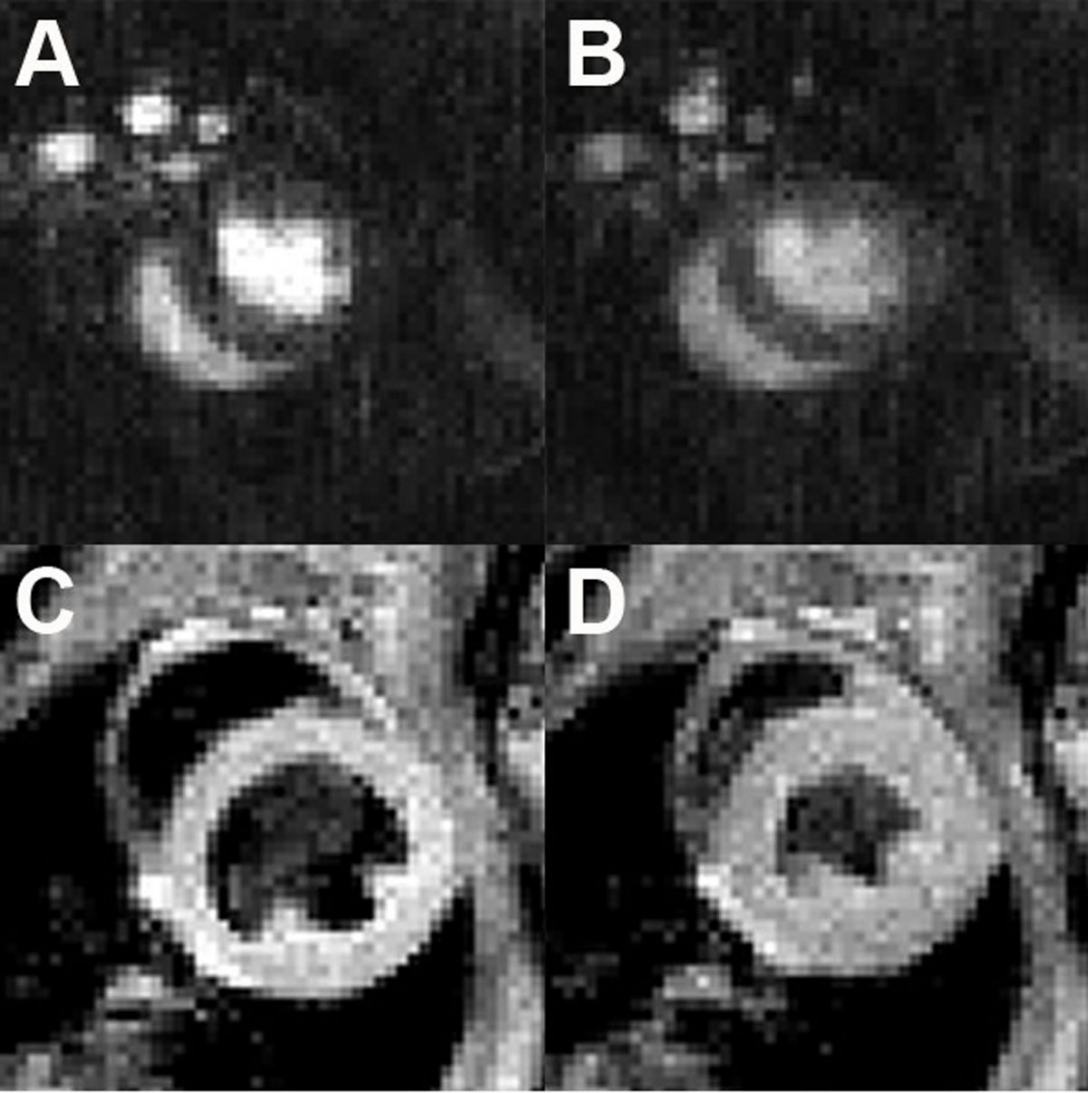
Example first-pass gadolinium-enhanced images (A-B), and cine images (C-D) of the mouse heart.

**Figure 2:**
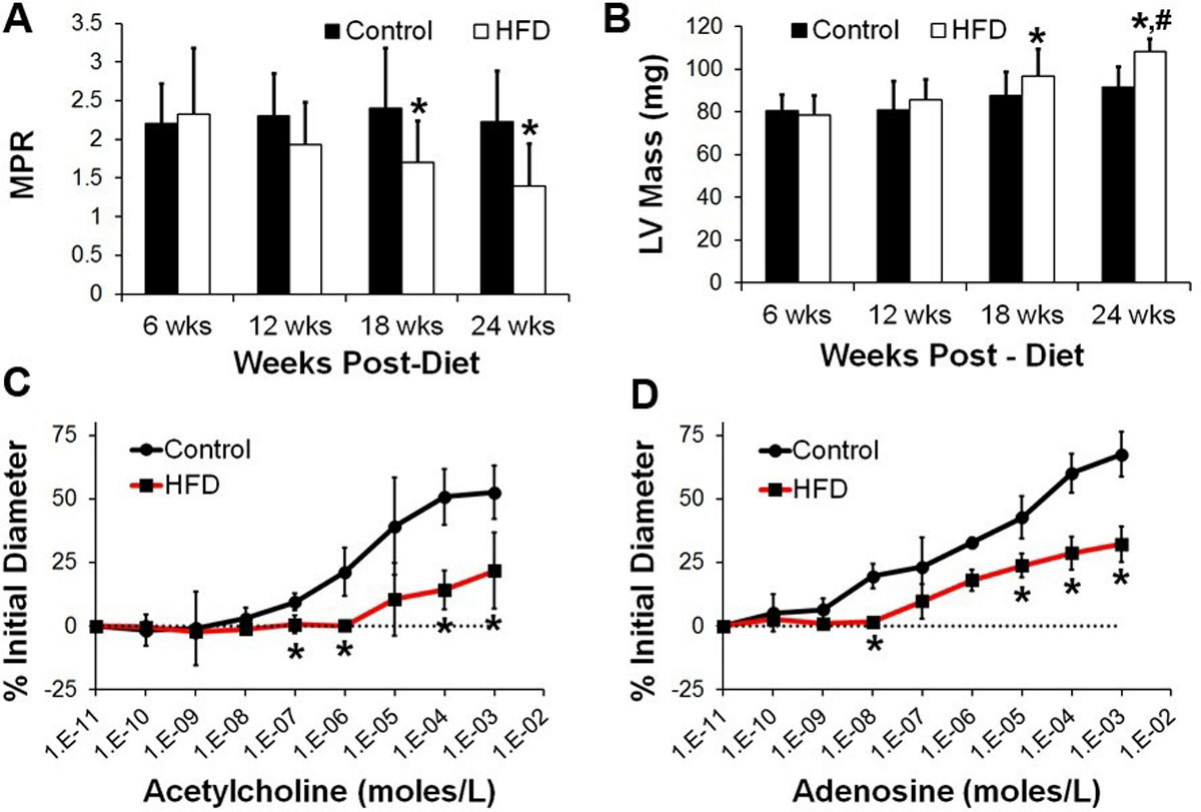
(A): MPR was reduced in the HFD mice as compared to the control mice at 18 and 24 weeks after diet (*p<0.05 vs. age-matched control). (B): LV mass was increased in HFD mice at 18 wks after diet (*p<0.05 vs. control) and it further increased at 24 wks after diet (*p<0.05 vs. control, #p<0.05 vs. HFD at 18wks). (C): Cumulative dose responses of coronary arterioles to acetylcholine. HFD mice had reduced ability to dilate in response to acetylcholine (*p <0.05 vs. control). (D): Cumulative dose responses of coronary arterioles to adenosine. The ability to dilate the coronary arterioles in response to adenosine is significantly inhibited in the HFD mice (*p<0.05 vs. control).

HFD mice were obese relative to Control mice at 6 weeks of diet (31.8±5.8g vs. 24.5±5.2g, p<0.05) and their body weight progressively increased up to 24 weeks post-diet (45.5±5.3g vs. 30.6±4.4g, p<0.05). Figure 1 shows examples of first-pass perfusion MRI (A-B) and cine MRI (C-D) from a mouse heart. MPR in HFD mice was reduced at 18 and 24 weeks post-diet (Figure 2A, p<0.05 vs. age-matched control). LV mass was increased in HFD mice at 18 weeks (p<0.05 vs. Control) and it further increased at 24 weeks (Figure 2B, p<0.05 vs. Control, HFD at 18 weeks). LV wall thickness was increased in HFD mice at 18 and 24 weeks post-diet (p<0.05 vs. age-matched Control). Vascular reactivity of the coronary arterioles in response to acetylcholine (Figure 2C) and adenosine (Figure 2D) was reduced in HFD mice (p<0.05 vs. Control). There were no significant differences in volume, ejection fraction, blood pressure and capillary density measurements between the two groups. Histology showed no aortic atherosclerosis in HFD or Control mice.

## Conclusions

Using cardiac MR, vascular reactivity, and histological studies, we showed that C57Bl/6 mice fed a HFD for 18-24 weeks have LV hypertrophy and reduced vasodilatory capacity at stress with normal capillary density and no aortic plaque. Future studies using cardiac MR and gene-modified mice fed a HFD may shed light on key molecular mechanisms that underlie myocardial ischemia in obesity related cardiomyopathy.

## Funding

This work was funded by NIH R01 EB001763.
